# The predictive performance of surgical treatment in upper molars with combined bony defect and furcation involvement: a retrospective cohort study

**DOI:** 10.1186/s12903-022-02196-0

**Published:** 2022-05-06

**Authors:** Meng-Yao Chiu, Cho-Ying Lin, Pe-Yi Kuo

**Affiliations:** 1 Department of Periodontics, Chang Gung Memorial Hospital, No. 199, Tun Hua N. Rd., Taipei, 105 Taiwan; 2 Chang Gung University, 259 Wen-Hwa 1st Road, Kwei-Shan Tao-Yuan, Taoyuan City, 333 Taiwan

**Keywords:** Furcation involvement, Intrabony defect, Osseous resective surgery, Periodontal regeneration, Guided tissue regeneration, Residual pocket

## Abstract

**Background:**

To evaluate the impact of combined defects, bony destruction and furcation involvement, on disease resolution after surgery in terms of pocket elimination, absence of inflammation, furcation improvement and predictive performance.

**Methods:**

Combined bony (intrabony (+) or (−)) and furcation defects (FI degree 1 or 2) at maxillary molars in patients diagnosed as periodontitis stage III to IV, being through periodontal surgery and at least 6 months follow-up were retrospectively screened. Cumulative predictability (CR, %), failure of treatment and the change of clinical parameters from baseline at pre-operative visit to the latest maintenance care, including pocket depth (PD), horizontal and vertical furcation involvement (FI) were analyzed. Failure of treatment with low predictability was defined as residual PD > 4 mm with bleeding on probing during maintenance period.

**Results:**

Thirty-three patients with fifty-one combined defects were included. Statistical analysis showed significant overall PD reduction and FI improvement (*p* < 0.001). Combined FI degree 2 with intrabony (+) defects revealed more horizontal furcation improvement compared with FI degree 2 with suprabony defect (*p* = 0.007). However, type of combined defects was not relevant to CR (*p* = 0.702) and PD reduction (*p* = 0.707). Among all parameters, baseline PD with proximal FI degree 2 was indicated to failure of treatment.

**Conclusions:**

Different types of combined defects, deep baseline pocket and proximal FI degree 2 would compromise the predictability of treatment outcomes in upper molars. Nevertheless, the combination of surgical treatment and strict maintenance care could still yield high predictability and survival rate.

*Trial registration*: retrospectively registered.

**Supplementary Information:**

The online version contains supplementary material available at 10.1186/s12903-022-02196-0.

## Background

In well-controlled periodontitis, the ultimate goal of periodontal treatment is not only to cease progressive tissue breakdown but also to maintain the stable condition during follow-up period. To define the stable condition of diseased tooth, shallow pockets ($\le$ 4 mm) without bleeding on probing has been deemed as the endpoint of active periodontal treatment [1]. With the concomitant furcation involvement, irregular bony architecture and intrabony defects caused by periodontal destruction could be attributed to residual deep pockets, which might be a hazard to further tooth survival [2].

To eliminate the residual pockets and facilitate further maintenance care, different surgical modalities have been widely used [3]. Basically, the application of osseous surgery for bone recontouring and regenerative therapy for tissue regeneration are principle surgical options, and which one to be used mainly depends on defect configuration. Osseous surgery is an optimal treatment for shallow defects ($\le$ 3 mm) and bony deformities [4], while deep intrabony defects [5–8] and degree 2 furcation involvement [9, 10] could be benefited from regenerative therapy with different materials. However, following previous decision-making process, clinical failures with unsatisfied results still occurred. This could mainly be ascribed to the fact that actual defect morphology was more complicated than the ones in well-designed clinical trials, in which only pure supra- or intra-bony defects without furcation involvement were recruited [11–13]. Likewise, in the studies focusing on furcation treatment, absence of data about surrounding bony defects should also be mentioned [14–20].

The combination of supra- or intra-bony defects with furcation involvement could be commonly found at posterior dentition, and as a result using the same strategies in treating combined defects might fall short at the end. Since the results of surgical treatment in combined defects was sparsely discussed [21], a new classification of combined defects might be necessary to establish a more comprehensive treatment implication.

Following pervious decision-making process [4–9, 12–14, 22], the aim of this retrospective cohort study was to investigate the surgical outcomes based on different types of combined defects. Hence, the primary outcome was to assess the cumulative predictability (CR, %), failure of treatment and the change of clinical parameters, such as pocket depth (PD), vertical and horizontal furcation involvement (FI) from baseline at pre-operative examination and the latest follow-up visit. Furthermore, the secondary outcome was to evaluate all possible correlative factors determining predictability of treated sites, which was defined as pocket < 4 mm without bleeding on probing.

## Methods

### Patient population

The ethic approval of this study protocol was reviewed and approved by Chang Gung medical foundation institutional review board (IRB no.: 202101383B0) according to the Declaration of Helsinki on experimentation involving human subjects and the Belmont report. Strengthening the Reporting of Observational studies in Epidemiology (STROBE) statement [23] was also fully complied (Additional file 1: Form).

In this retrospective cohort study, subjects treated at department of periodontics in Taipei Chang Gung memorial hospital met the following inclusion criteria were recruited since 2016 January to 2020 June: (1) patients beyond 20 year-old was diagnosed with stage III to IV periodontitis [24] with more than one furcation involving maxillary molars, (2) patients had received both periodontal phase I therapy and surgical treatment, (3) Good compliers have been through routine follow-up for at least 6 months [25–28]. Exclusion criteria were: (1) heavy smokers (> 10 cigarettes per day), (2) with pregnancy and metabolism or immune diseases, (3) tooth diagnosed as hopeless prognosis [29] or with Miller’s grade III mobility [30], (4) any loss of follow-up visits, and (5) incomplete clinical and radiography data collection.

The sample size was determined based on previous study by Majzoub et al. [17], in which clinical outcomes and survival of furcated molars treated with guided tissue regeneration were analyzed. With a power of 80% and 5% of alpha-error, at least 38 samples in total were required to detect a difference of 2 mm in pocket depth change. The value of at least 2 mm of pocket depth change was used to prevent probing error suggested by Mombelli et al. [31].

### Clinical measurements

Data were collected at initial examination, re-evaluation visit (baseline), during surgery and at the most recent visit of supportive care by the same calibrated clinician (CYL). The following clinical parameters were evaluated: (1) PD was recorded at six aspects per tooth to the nearest millimeter (PCP-UNC 15 tip, Hu-Friedy, Chicago, IL). The deepest PD at proximal sites was selected for evaluation. (2) Bleeding on probing (BoP) and presence of plaque were recorded dichotomously at six probing sites, and the data was calculated in percentage as full-mouth bleeding scores (FMBS) and full-mouth plaque scores (FMPS). (3) Tooth mobility was assessed clinically and classified according to Miller scale [30]. (4) Degree of horizontal [32] and vertical [33] FI were recorded clinically and re-checked with vertical bitewing x-ray films during follow-up visits [34]. (5) Defect morphology was described and recorded during surgery and divided into suprabony and intrabony defects [35].

### Study design and surgical technique

All treatments were performed by the same clinician (CYL). After initial clinical and radiographic examination, all patients received non-surgical periodontal treatment including scaling, supragingival and subgingival debridement and oral hygiene instruction. Re-evaluation visit was arranged 2 months later. Sites with residual PD more than 5 mm, bleeding on probing and bony defect showed on x-ray were scheduled for periodontal surgery. Prior to surgical treatment, all patients had full-mouth plaque scores and full-mouth bleeding score below 20%. All surgical treatments were performed with the aid of loupe (× 3.8) for better visibility and accessibility. Following local anesthesia, simplified or modified papilla preservation flaps and a thorough debridement with curettes (Gracey, Hu-Friedy, Chicago, IL, USA) and ultrasonic device (Universal Insert 25 K Internal water ultrasonic scaler, Dentamerica) were performed.

**Fig. 1:**
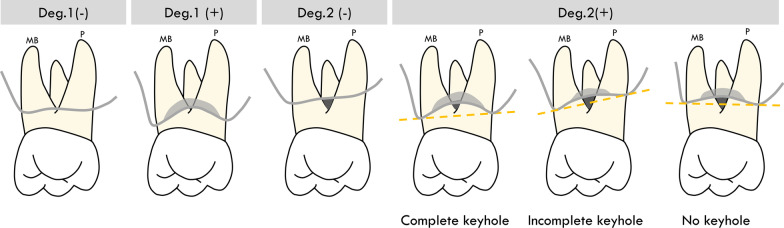
Schematic diagram of different groups. Deg., degree

Defect morphology were recorded, and a new classification of combined defects with four groups was well defined according to degree of horizontal furcation involvement [32] and types of bony defect [35] as following (Fig. 1):

- Deg.1(−): degree 1 furcation involvement with suprabony defect
- Deg.1(+): degree 1 furcation involvement with intrabony defect
- Deg.2(−): degree 2 furcation involvement with suprabony defect
- Deg.2(+): degree 2 furcation involvement with intrabony defect

Depending on defect configurations, different regenerative therapy strategies were performed, including guided tissue regeneration with resorbable collagen membrane (OsseoGuard®, Zimmer Biomet, USA), regenerative therapy with enamel matrix deveriates (EMD) (Emdogain®, Straumann®, Basel, Switzerland) and combination therapy by using either GTR or EMD with alloplasts (Sinbone HT®, Purzer Pharmaceutical, Taipei, Taiwan) or xenografts (Bio-Oss®, Geistlich Pharma AG, Wolhusen, Switzerland). When treating contained defects with degree I-II FI and 2- to 3- wall intrabony components, regenerative treatment was used, and resective surgery, such as odontoplasty, osseoplasty or osseotectomy would be performed in non-contained 1-wall components in terms of wound stability. The wound was sutured with 4-0 Vicryl for primary closure. Wound dressing (Coe-Pak, GC, USA) was placed and left for 2 weeks before suture removal. Acetaminophen tablets (500 mg) Q6H and amoxicillin capsules (500 mg) tid for at least three days were prescribed to prevent post-surgery pain and infection. Any use of toothbrushing devices was forbidden around surgical sites for 14 days post-surgery. Thereafter, strict supportive periodontal treatment (SPT) was performed in every 3- to 4- months supportive care visits, including supra- and sub-gingiva prophylaxis, oral hygiene instruction and reinforcement with toothbrush, interdental brush and end-tufted brush.

### Clinical outcomes, characteristics of failed cases and predictability

The primary outcome was to evaluate the predictive performance of surgical treatment, including changes of FI (both horizontal and vertical), PD reduction and specifically the alteration of proximal furcation. According to clinical observation and histologic evidence in related studies, tissue healing period would gradually slowed down 6 months following osseous surgery [4], and new bone, cementum and periodontal ligament formation could be detected 6 months after regenerative therapies [25–28]. Considering that neither non-FI defects nor FI degree I would not increase the risk of tooth loss under strict SPT program [14, 36–38], “furcation improvement” in present study indicated complete furcation closure or conversion to degree 1. Besides, treated sites with residual PD > 4 mm and concomitant BoP were assessed as failed cases, and the characteristics of the failing population were further assessed. On the contrary, “predictability” was defined as PD less than 4 mm without BoP [1] over time. In order to consider the time factor, the predicative performance of each site was presented with the percentage of “cumulative predictability” (CR, %), and the comparisons were conducted between FI degree (1, 2) [32], subclassification (A, B, C) [33] and the classification of 4 types of combined defects based on (Deg.1(−)/(+), Deg.2(−)/(+)). Besides, predictability more than 70% was regarded as high, and 50–70%, less than 50% were seen as moderate and low respectively. All the data was collected and screened by one independent reviewer (MYC), and the superior reviewers (PYK, CYL) would make the final decision in agreement when confronting uncertain cases.

### Statistical analysis

Descriptive statistics were recorded as mean values with standard deviation (meant $\pm$ SD). Shapiro–wilk analysis was used for distribution analysis of PD, horizontal and vertical FI at baseline. Wilcoxon signed-rank test was conducted to identify changes of horizontal and vertical FI as well as the change of PD from baseline to the latest follow-up visit. Subgroup analysis were calculated with Mann-Whiney and Kruskal–Wallis test. Spearman's Rho analysis was used to find correlation between the grade of periodontitis, horizontal FI degree and baseline PD. Univariate logistic regression was used to evaluate possible factors related to failure cases. Kaplan Meier curve was utilized to detect the predictability over time according to defect types, the degree of horizontal and vertical FI. The statistical significance of P value was set at 5%. All collected data and statistical analysis were documented and performed with SPSS (IBM SPSS Statistics for Mac, Version 25.0. Armonk, NY: IBM Corp) and JASP statistical software (Version 0.14.1.0).

## Results

### Study characteristics

**Fig. 2:**
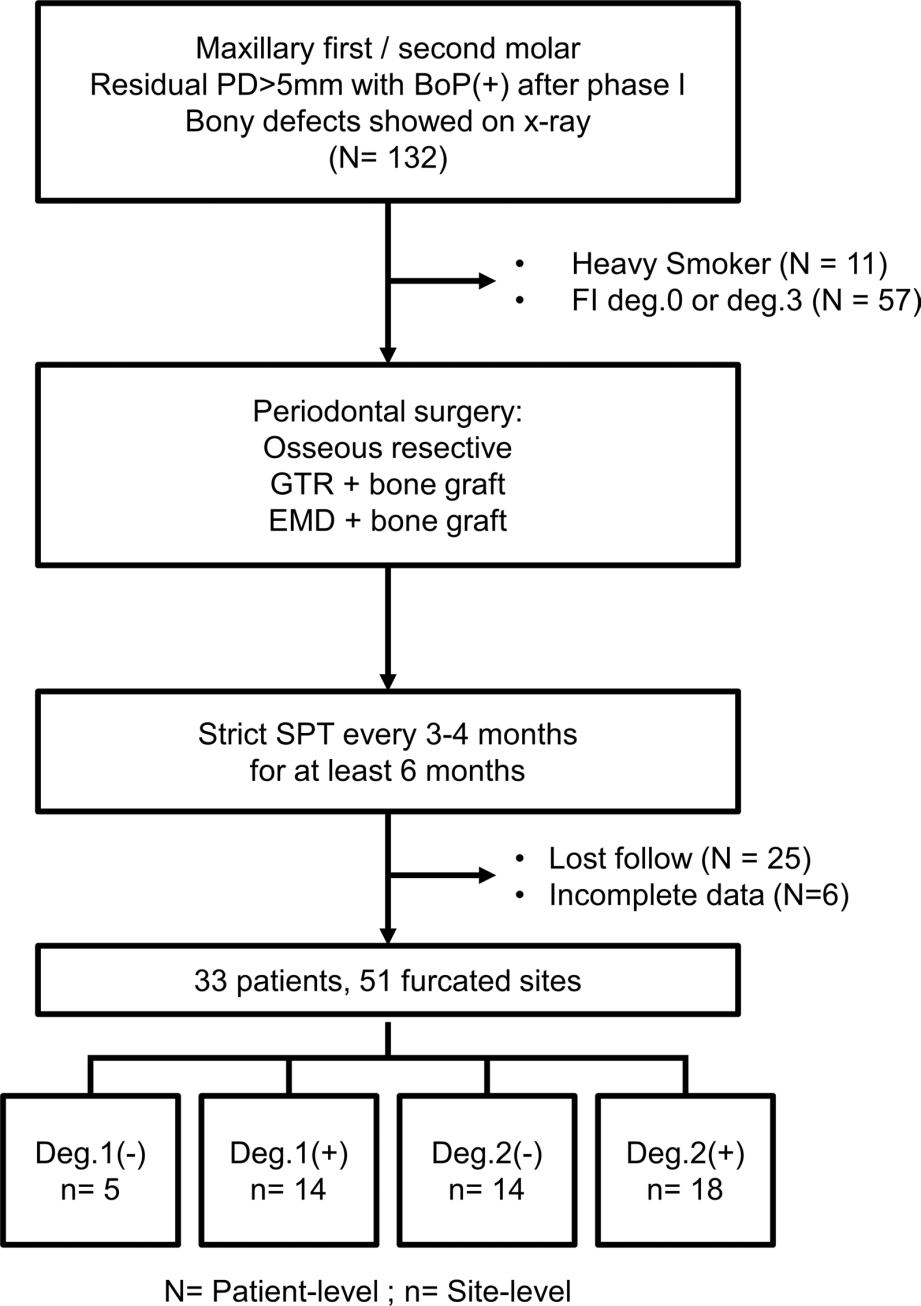
Flow chart of this study. PD, pocket depth; BoP, bleeding on probing; Deg., degree; GTR, guided tissue regeneration; EMD, enamel matrix derivative; SPT, supportive periodontal treatment; N, number (patient level); n, number (site level)

After thorough screening, 99 patients were excluded from this study due to heavy smokers (N = 11), with FI degree 0 or 3 (N = 57), erratic compliance (N = 25) and incomplete data collection (N = 6) (Fig. 2). Finally, 33 patients (23 female and 10 male) with 43 teeth and 51 combined defects were included. Table 1 illustrated the characteristics at patient/site level and the comparison between different defect groups. Mean age of subjects was 50.5 ± 8.1 years old. The mean duration of follow-up time was 25.4 ± 14.8 months. Among all patients, 69.7% (N = 23) were diagnosed as periodontitis stage III, grade B and the remaining 30.3% patients (N = 10) were stage III, grade C. Regarding surgery modalities, 25.5% (n = 13) were treated with osseous surgery, 39.2% (n = 20) with GTR and allograft, and 35.3% (n = 18) with EMD and allograft. All tooth survived after surgical treatment, and all treated sites healed uneventfully without wound dehiscence or membrane exposure. As for site-specific data, Shapiro–wilk analysis showed that baseline horizontal FI, vertical FI and PD were not normally distributed, and Kruskal–Wallis test was used for nonparametric statistics to analyze the differences of baseline PD and PD change between groups. There was no significant difference in PD at baseline and the change of PD among 4 groups of different combined defects (Table 1). No correlation between stage/grade system of periodontitis, PD and horizontal FI at baseline was found (Spearman's Rho correlation, *p* > 0.05).

**Table 1:** Characteristics and results of the included patients/defects

*Patient-level data*				
Patient (N)	33			
Gender (female/male) (N) (%)	23 (69.7%) / 10 (30.3%)			
Age (years)	50.5 $\pm$ 8.1			
Periodontitis stage & grade (N) (%)	Stage III, Grade B: 23 (69.7%) Stage III, Grade C: 10 (30.3%)			
*Site-level data*				
Tooth on surgery (N)	43			
Tooth survival (%)	100%			
Furcation sites per group (N)	Deg.1(−)	Deg.1(+)	Deg.2(−)	Deg.2(+)
	5	14	14	18
Tooth location (first/second molar) (N)	2/3	3/11	9/5	11/7
Follow-up period (months)	27.8 $\pm$ 18.1	28.2 $\pm$ 11.0	22.0 $\pm$ 12.1	25.1 $\pm$ 18.0
Furcation horizontal change (improvement/no change/deterioration) (N)^*^	4/1/0	10/4/0	8/6/0^**^	17/1/0^**^
Furcation vertical change (improvement/no change/deterioration) (N)^*^	4/1/0	12/2/0	11/3/0	15/2/1
Baseline PD (mm)^***^	5.80 $\pm$ 0.84	6.57 $\pm$ 1.02	6.21 $\pm$ 1.67	6.94 $\pm$ 1.51
Post-OP PD (mm)	3.40 $\pm$ 1.14	3.79 $\pm$ 0.89	4.00 $\pm$ 0.88	4.17 $\pm$ 1.10
PD reduction (mm)^*, ***^	2.40 $\pm$ 1.67	2.79 $\pm$ 1.25	2.21 $\pm$ 1.48	2.78 $\pm$ 1.66
Success (N, %)	4 (80%)	11 (78.6%)	9 (64.3%)	12 (66.7%)
Failure (N, %)	1 (20%)	3 (21.4%)	5 (35.7%)	6 (33.3%)

N, number; PD, pocket depth; Deg, degree

*Significantly different change from baseline, *p* < 0.0001 (Wilcoxon signed-rank test)

**Significantly different between groups, *p* = 0.007 (Mann-Whiney test)

***No statistically significant difference between groups, *p* > 0.05 (Kruskal–Wallis test)

### Surgical outcomes in combined defects

**Fig. 3:**
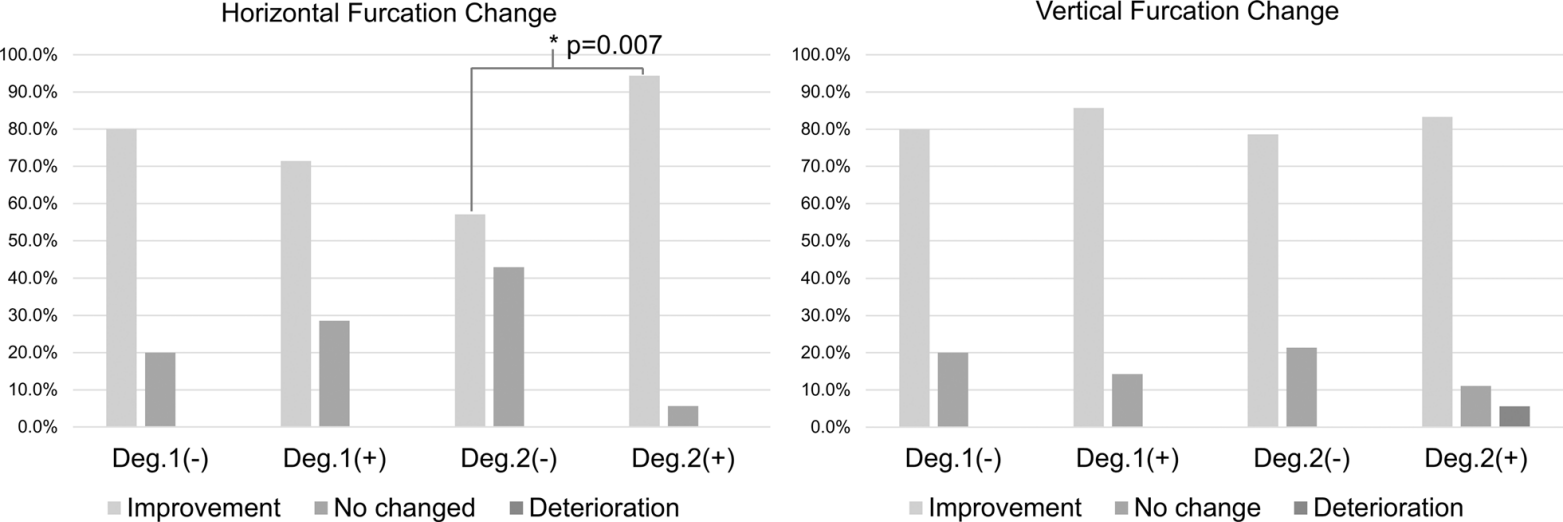
Surgical outcomes of horizontal and vertical furcation involvement in different groups. Deg., degree. ^*^Statistical significance

**Fig. 4:**
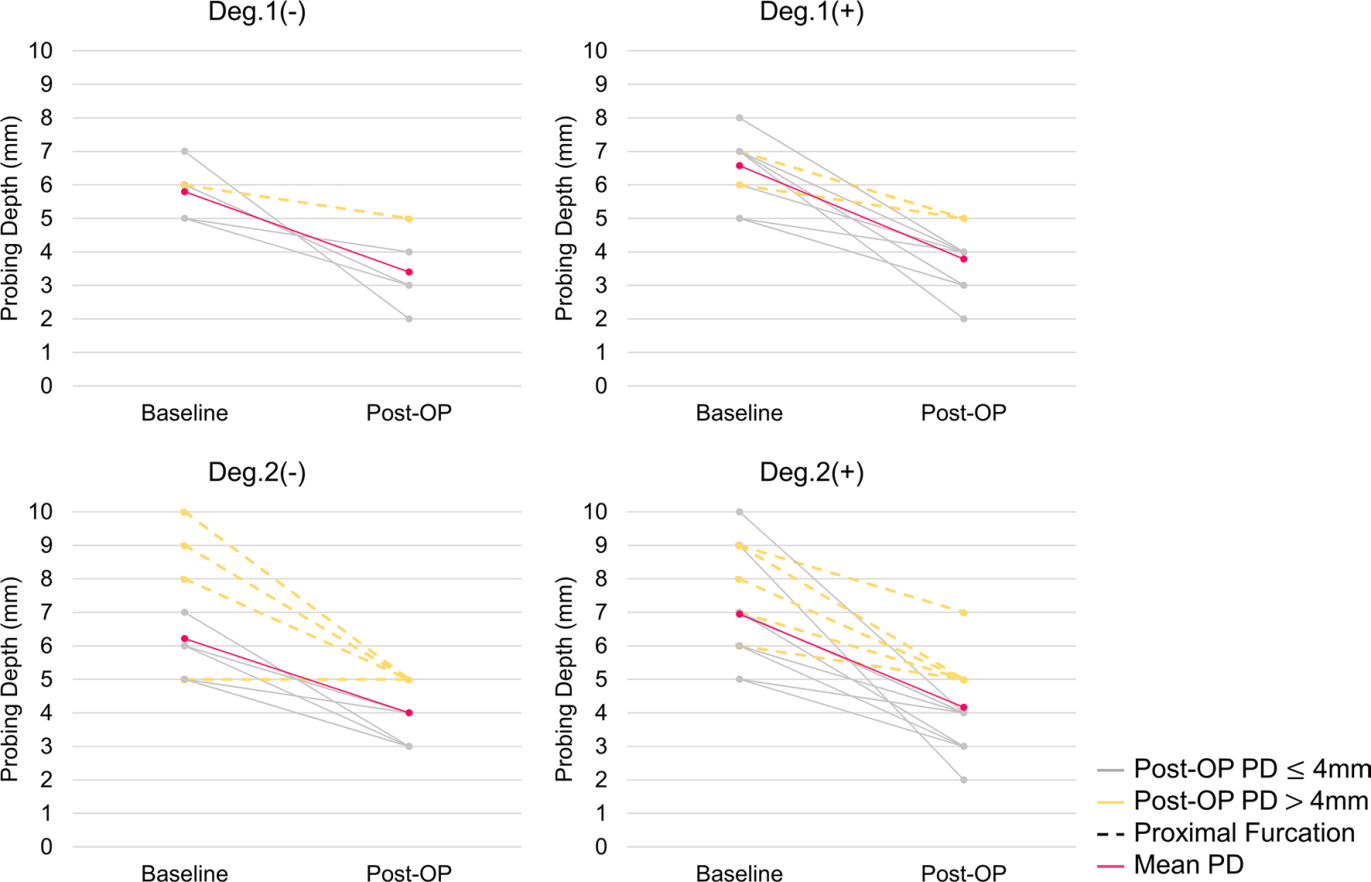
Changes of pocket depth in different groups. PD, pocket depth; Deg., degree

As for surgical outcomes, both horizontal and vertical FI had significant improvement after surgery (*p* < 0.001). Improvement of horizontal FI showed 80.0% (N = 4), 71.4% (N = 10), 57.1% (N = 8) and 94.4%(N = 17) in group Deg.1(−), Deg.1(+), Deg.2(−) and Deg.2(+) respectively, and significant difference between group Deg.2(−) and Deg.2(+) (*p* = 0.007) could be observed. However, no statistically significance was found between groups in view of vertical FI (Table 1, Fig. 3). In site-specific perspective, mean PD reduction was 2.56 ± 1.49 mm with significant improvement from baseline (*p* < 0.001) but no statistical difference between groups (*p* = 0.693, Table 1, Fig. 4). Out of total 51 defects, 15 treated sites had residual PD more than 4 mm with BoP, which showed 20–35.7% failure rate in each group (Table 1).

**Fig. 5:**
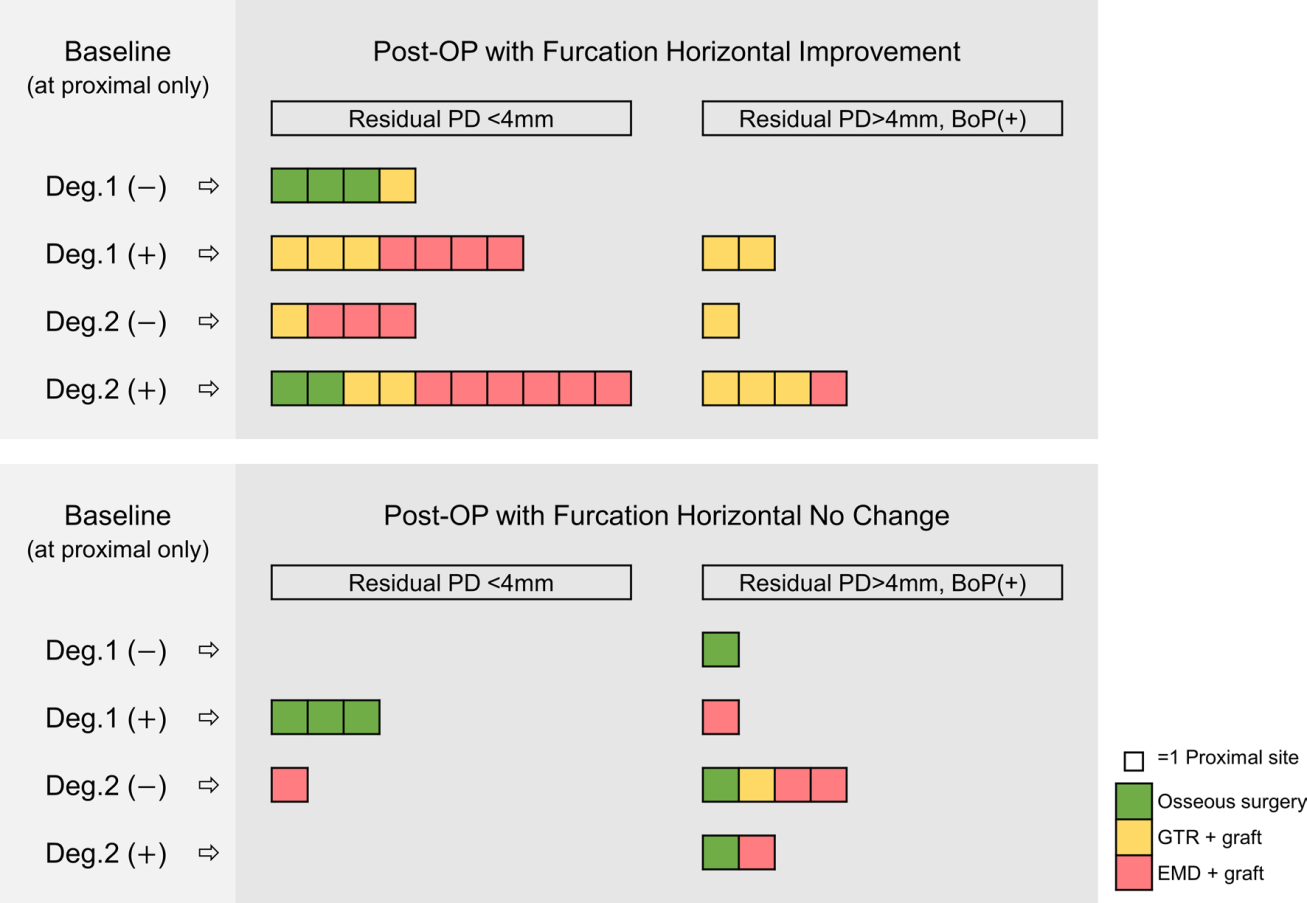
Distribution of surgical modalities and treatment outcomes in proximal area

Treated sites with PD > 4 mm and BoP following surgical treatment were listed in Additional file 2: Table S1 with clinical descriptions. All failed sites were at proximal areas, and 73.3% (N = 11) of all had degree 2 FI with deeper initial PD (Additional file 2: Table S1, Fig. 4). Focusing on proximal areas, residual PD with BoP could still be found following regenerative therapy, and the application of osseous surgery could more frequently be found with PD less than 4 mm. (Fig. 5). Univariate logistic regression analysis showed that the only factor associated with failed cases was baseline PD (odd ratio 1.634; 95% CI 0.947–0.035; *p* = 0.035). In contrast, other variables, including groups with different defects, periodontitis grade, and horizontal and vertical FI were not statistically correlated (Table 2).

**Table 2:** Univariate logistic regression to evaluate possible factors related to failure

Variable	Estimate	Standard error	Odd ratio	95% CI	*p* value
Group—reference: Deg.1(−)					
Deg.1(+)	0.087	1.294	1.091	− 2.449, 2.623	0.946
Deg.2(−)	0.799	1.249	2.222	− 1.650, 3.247	0.523
Deg.2(+)	0.693	1.225	2.000	− 1.707, 3.094	0.571
Grade—reference: Grade B					
Grade C	0.223	0.785	1.250	− 1.316, 1.762	0.776
Horizontal furcation—reference: horizontal furcation involvement degree 1					
Degree 2	0.675	0.675	1.964	− 0.647, 1.997	0.317
Vertical furcation—reference: vertical furcation subclassification A					
Subclass B	0.827	0.680	2.286	− 0.506, 2.159	0.224
Subclass C	0.827	1.009	2.286	− 1.151, 2.804	0.413
Baseline pocket depth					
	0.491	0.233	1.634	0.035, 0.947	0.035*

Deg, degree

*Statistical significance

### Predictive performance of different groups over time

**Fig. 6:**
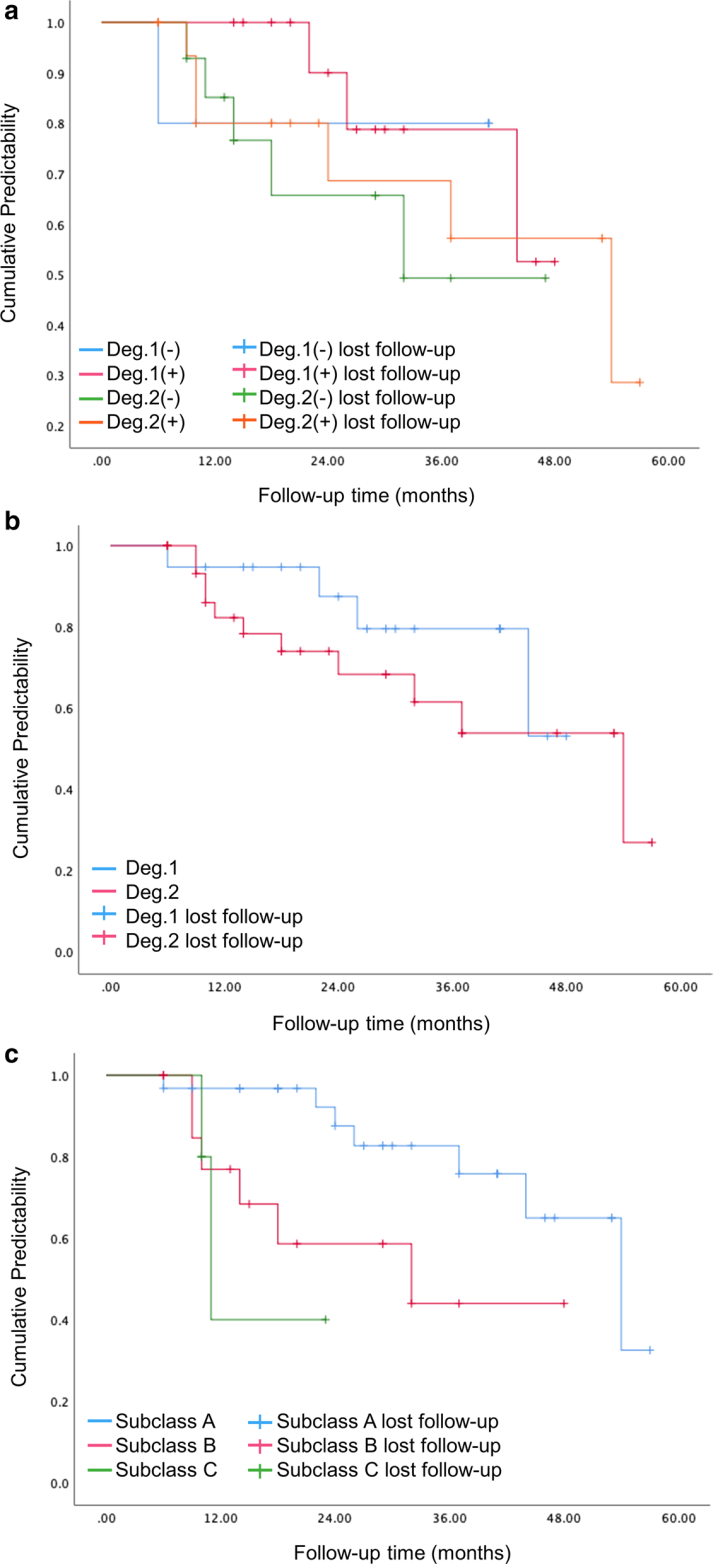
Kaplan–Meier curves of cumulative predictability according to **a** groups, **b** horizontal furcation degree, **c** vertical furcation degree. Deg., degree

Based on Kalpan Meier curve analysis for predictive performance, higher degree of horizontal and vertical FI at baseline were negatively associated with cumulative predictability, but statistically difference was only showed in vertical component (*p* = 0.026) (Fig. 6b, c). In general, high predictability was presented in all groups, and the favorable outcomes could last only in Deg. I(+)/(−) groups after 12 months. (Figure(a)). Despite statistically significance was lacking, Deg.2(−) defects had low predictability after 36-month follow-up, followed by moderate, high, high status in Deg.2(+), Deg.1(+) and Deg.1(−) respectively.

## Discussion

Difficulty in accessibility of oral hygiene maintenance has deem as the main reason of periodontal destruction particularly in posterior teeth [9, 39, 40], especially in maxillary molars with the presence of trifurcations [41–43]. As for tooth survivability, teeth with residual pockets, bleeding tendency and furcation invasion might pose a higher risk of failing supportive treatment [2, 34, 44]. Besides, it might be unpractical to search for the superior approach among all surgical treatments because of the possible limitations: tooth proximity, presence of key- hole defect, patient compliance, accessibility of daily care and duration of follow-up period. In addition, available evidence was mostly from pure defect type, and the result in combined defects, including FIs and bony defects, was sparsely discussed. Therefore, the present study tried to evaluate the impacts of combined defects with surgical outcomes during maintenance care.

### Surgical modalities and clinical outcomes in combined defects

Resective surgery, including osseous surgery [4, 13] and furcoplasty [32] could be one of the valid means in pocket elimination, furcation reduction and better oral hygiene care. On the other hand, regenerative therapy with biomaterials have shown promising regeneration potentials [6, 8] and reduced PD during maintenance period [45]. However, regenerative outcomes in furcation lesion could be unpredictable sometimes. Additionally, most of the favorable results were exclusively presented in buccal FI degree 2 of lower molars [9, 14], proximal furcation defects might be more challenging due to difficulties in instrument accessibility and unstable barrier membrane adaptation [46, 47]. Even though using EMD with bone grafts might offer some benefits in proximal furcations, the outcomes were still diverse between studies [48, 49]. In the present study, the application of osseous surgery with regeneration could improve the surgical outcomes in combined defects with proximal furcations, which implied the importance of adequate space maintenance in proximal combined defects during healing process.

In agreement with previous study [50], favorable outcomes in FI improvement with 100% survival rate could be obtained after surgery and strict SPT in current study, while intergroup differences in clinical outcomes should be noted. Intergroup analysis revealed that Deg.2(+) defects had statistically more horizontal furcation improvement (*p* = 0.007) than Deg.2(−) defects, which could be ascribed to the potential of space maintenance in contained defects around furcation lesions. Additionally, no significant difference was found in vertical component, and the possible explanation could be lack of complete “key-hole” defect morphology in most furcation defects [10]. Comparing to one recent study with combined defects, less favorable improvement in degree 2 FI (81.25% versus 95%) was obtained in present study [21], and the possible reason was that it only included defects with interproximal bone at the level or coronal to the furcation entrance. However, key-hole defect with high proximal bone level could not be anticipated in all clinical circumstances.

### Clinical characteristics of failed sites

It was also worth noting that most failed sites with residual PD located at proximal areas with FI degree 2. Among all possible predictors to failing cases, PD before surgery was the only one determining the outcomes, and the value might generally depict the severity of periodontal destruction. In line with previous studies, deep PD at baseline had been regarded as a negative factor to furcation closure [10] and tooth survival [37]. Nevertheless, results from this study were not able to correlate PD at baseline with residual PD, regeneration breakdown in furcations and tooth loss due to limited sample sizes, no re-entry surgery and short-term follow-up.

### Predictive performance over time in different groups: rate and tendency

Regarding the predictive performance of treated sites, tooth loss as the endpoint was not adequate for short to medium follow-up (< 5 ~ 10 years), the present study utilized the degree of cumulative predictability over time to depict the alteration tendency in different conditions. According to Kaplan Meier curve of predictability, horizontal and vertical furcation degree was potentially proved as dose-dependent effects, which concurred with the tendency in tooth loss with long-term observation [34, 36, 37, 44, 51]. Within 12-month follow-up, the overall predictability was high in all groups, while the advantage could not be held in degree2(+)/(−) defects after then. Focusing on deg.2 groups, the noteworthy findings indicated better predictability in deg.2(+) defects compared with deg.2(−), and the discrepancy even increased with time after 36 months. However, the difference did not reach significance due to limited sample size and follow-up time.

### Clinical implications and limitations

Since residual PD with severe FI might be responsible for tooth loss during maintenance period [2, 36, 37, 44, 51, 52], strict SPT with plaque control is mandatory. Several retrospective studies have concluded that frequency of supportive care and patient compliance had tremendous influence on long term stability after regeneration [53–55]. Annual tooth loss rate could be doubled in non-maintained group [56]. In addition, increased risk of tooth loss was found in erratic compliers with furcation involvement [37, 38]. Consequently, consistent maintenance care following surgical treatment is essential for long term stability.

Limitations of this study were its retrospective nature, small sample sizes in each group and only short to medium term follow- up period. Hence, comparisons between different treatment modalities and long-term survival rate were not available. Moreover, aside from the change of PD, data of horizontal and vertical clinical attachment level might also be required to demonstrate the regeneration outcomes. Regarding the combined defects, both FI and adjacent destruction, were particularly focused in present study, the descriptions of defect configuration in detail should be mentioned for further investigation. Therefore, well designed prospective studies with intact data and long-term follow-up would be necessary in future to search for a more predictable therapeutic strategy in combined defects.

## Conclusion

Based on the results of this study, with a mean 24-month follow-up, different types of combined defects did have impacts on predictive performance of treatment outcomes, and Deg.2(+) lesions was more predictable in furcation improvement, shallow PD and absence of BoP under strict supportive treatment. Regardless of different surgical modalities, deep PD at baseline was a crucial predictor in failed cases. Nevertheless, high predictability (> 70%) and survival rate (100%) of overall outcomes in upper molars with combined defects could still be obtained by means of surgical treatment and maintenance care.

## Supplementary Information

**Additional file 1**. Strengthening the Reporting of Observational studies in Epidemiology (STROBE) statement. Checklist of items that should be included in reports of cohort studies.

**Additional file 2: Table S1**. Detail information of all these failed sites. Abbreviations: CEJ-bot, distance from cementoenamel junction to defect bottom; CEJ-crest, distance from cementoenamel junction to bone crest; Deg, degree; GTR, guided tissue regeneration; EMD, enamel matrix derivative.

## Data Availability

The datasets generated and/or analyzed during the current study are not publicly available due Chang Gung Memorial Hospital medical record collection and privacy policy but are available from the corresponding author on reasonable request.

## Abbreviations

BoP: Bleeding on probing
CEJ-bot: Distance from cementoenamel junction to defect bottom
CEJ-crest: Distance from cementoenamel junction to bone crest
CI: Confidence interval
CR: Cumulative predictability
Deg.: Degree
EMD: Enamel matrix derivative
FMBS: Full-mouth bleeding scores
FMPS: Full-mouth plaque scores
FI: Furcation involvement
GTR: Guided tissue regeneration
N: Number (patient level)
n: Number (site level)
PD: Pocket depth
SD: Standard deviation
SPT: Supportive periodontal treatment
