# Supplementary material for: The predictive performance of surgical treatment in upper molars with combined bony defect and furcation involvement: a retrospective cohort study
Source: BMC Oral Health. 2022 May 6;22:156. doi: 10.1186/s12903-022-02196-0 (PMC9074367; doi:10.1186/s12903-022-02196-0)
Supplement: Supplementary file 2 — Additional file 2: Table S1. Detail information of all these failed sites. Abbreviations: CEJ-bot, distance from cementoenamel junction to defect bottom; CEJ-crest, distance from cementoenamel junction to bone crest; Deg, degree; GTR, guided tissue regeneration; EMD, enamel matrix derivative. [file 12903_2022_2196_MOESM2_ESM.docx]

**TABLE S1** Detail information of all these failed sites.

| Patient No. | Site | Defect description | Post-OP FI | Baseline PD  (mm) | Post-OP PD  (mm) | Treatment |
| --- | --- | --- | --- | --- | --- | --- |
| 4 | 27 mesial | - Deg.2(+), subclass A - Circumferential 3-wall defect, 2mm in depth, CEJ-bot = 7mm (>50%) - Key hole (-) | Deg.1, subclass B | 9 | 7 | GTR + bone graft |
| 8 | 16 mesial | - Deg.2(+), subclass A - 1-2 wall defect, 3 mm in depth and 3-4 mm in width, CEJ-bot =5mm - Key hole (-) | Deg.1, subclass 0 | 6 | 5 | GTR + bone graft |
| 10 | 26 distal | - Deg.2(+), subclass A - 1-2 wall defect, CEJ-bot = 5mm - Key hole (-) | Deg.0, subclass 0 | 8 | 5 | EMD + bone graft |
| 11 | 17 distal | - Deg.1(+), subclass A - 3-wall defect, 2-3 mm in depth and 3 mm in width, CEJ-bot = 3-4mm | Deg.0, subclass 0 | 7 | 5 | GTR + bone graft |
| 15 | 16 mesial | - Deg.2(+), subclass C - 2-3 wall defect, 4-5 mm in depth and 3 mm in width, CEJ-bot = 8mm - Key hole (+) | Deg.1, subclass 0 | 7 | 5 | GTR + bone graft |
| 18 | 27 distal | - Deg.2(+), subclass B - 1-2 wall defect, 3mm in width, CEJ-bot = 7mm - Key hole (-) | Deg.2, subclass A | 9 | 5 | Osseous surgery |
| 19 | 27 distal | - Deg.2(-), subclass B - CEJ-bot = 5mm, furcation divergence > 4mm - Key hole (-) | Deg.2, subclass A | 9 | 5 | GTR + bone graft |
| 22 | 26 distal | - Deg.2(+), subclass B - 1-2 wall defect, CEJ-bot = 7mm - Key hole (-) | Deg.2, subclass A | 6 | 5 | EMD + bone graft |
| 23 | 17 distal | - Deg.2(-), subclass B - Supra-eruption - Key hole (-), | Deg.2, subclass A | 5 | 5 | EMD + bone graft |
| 23 | 26 mesial | - Deg.2(-), subclass B - CEJ-bot = 6mm - Key hole (-) | Deg.2, subclass A | 5 | 5 | EMD + bone graft |
| 29 | 26 mesial | - Deg.1(+), subclass A - 2-wall defect, 3-4mm in depth, 2-3mm in width, CEJ-bot = 3mm - Key hole (-) | Deg.0, subclass 0 | 7 | 5 | GTR + bone graft |
| 31 | 27 distal | - Deg.1(+), subclass A - 2-3wall defect 2 mm in depth, 2 mm in width - Key hole (+) | Deg.1, subclass A | 6 | 5 | EMD + bone graft |
| 32 | 16 mesial | - Deg.2(-), subclass B - CEJ-crest = 6 - 7mm - Key hole (-) | Deg.1, subclass B | 8 | 5 | GTR + bone graft |
| 34 | 27 distal | - Deg.1(-), subclass A - CEJ-crest =3 mm - Key hole (-) | Deg.1, subclass A | 6 | 5 | Osseous surgery |
| 35 | 16 distal | - Deg.2(-), subclass C - Key hole (-) | Deg.2, subclass B | 10 | 5 | Osseous surgery |

Abbreviations: CEJ-bot, distance from cementoenamel junction to defect bottom; CEJ-crest, distance from cementoenamel junction to bone crest; Deg, degree; GTR, guided tissue regeneration; EMD, enamel matrix derivative
